# Discussion of Teaching With Multiple Intelligences to Corporate Employees' Learning Achievement and Learning Motivation

**DOI:** 10.3389/fpsyg.2021.770473

**Published:** 2021-10-18

**Authors:** Di-Yu Lei, Jui-Hsi Cheng, Chih-Ming Chen, Kai-Ping Huang, Chiyang James Chou

**Affiliations:** ^1^Fuzhou University of International Studies and Trade, Fuzhou, China; ^2^College of Business and Management, Xiamen Huaxia University, Fuzhou, China; ^3^School of Business, Fuzhou Institute of Technology, Fuzhou, China; ^4^Department of Business Administration, Social Enterprise Research Center, Fu Jen Catholic University, New Taipei City, Taiwan; ^5^Master Program in Entrepreneurial Management, National Yunlin University of Science and Technology, Yulin, Taiwan

**Keywords:** multiple intelligences, learning achievement, learning motivation, content-based curriculum, situated learning

## Abstract

The development of multiple intelligences used to focus on kindergartens and elementary schools as educational experts and officials considered that the development of students' multiple intelligences should be cultivated from childhood and slowly promoted to other levels. Nevertheless, the framework of multiple intelligences should not be simply promoted in kindergartens and elementary schools, but was also suitable in high schools, universities, and even graduate schools or in-service training. Taking employees in Southern Taiwan Science Park as the research subjects, total 314 employees in high-tech industry are preceded the 16-week (3 h per week for total 48 h) experimental teaching research. The research results show that (1) teaching with multiple intelligences would affect learning motivation, (2) teaching with multiple intelligences would affect learning achievement, and (3) learning motivation reveals remarkably positive effects on learning achievement. According to the results to proposed discussions, it is expected to help high-tech industry, when developing human resource potential, effectively well-utilize people's gifted uniqueness

## Introduction

Domestic education system, for a long time, paid attention to intellectual education. In the reflection before education reform, it was discovered that over-emphasizing intellectual education resulted in many students being sacrificed under the education system. Under the education reform in past years, the situation is gradually improved. Everyone possesses distinct intelligences and various combination and application methods that multi-methods should be used for the evaluation. Such methods provide special children with the growth model to develop the potential. Teachers' teaching with multiple intelligences allows such students fully developing the potential. Multiple intelligences particularly emphasize the application of intelligence in real life situations that the integration of teaching with multiple intelligences could help teachers assist in students' learning with multiple instruction and students expand abilities beyond subjects emphasized in traditional education. It would help current teaching styles.

Everyone presents the unique operation method that, with proper encouragement and guidance, the intelligence could achieve certain standards. For this reason, multiple intelligences allow each student finding out the sky and reaching the goal of adaptive development. The emergence of knowledge-based economy in past years reveals the importance of human capital of a nation. In face of increasing employment population domestically, understanding the ability for the right job in the right place is an extremely important issue for individuals or enterprises. The development of multiple intelligences used to focus on kindergartens and elementary schools as educational experts and officials considered that the development of students' multiple intelligences should be cultivated from childhood and slowly promoted to other levels. For high and elementary school students, multiple intelligences could help teachers better understand students from the intelligence distribution of students. For instance, multiple intelligences could be utilized for digging out gifted students and further providing them with suitable development opportunities to make the growth. Besides, multiple intelligences could be used for supporting students with problems and adopting more suitable methods for their learning. Regarding research on multiple intelligences, Ronald et al. (2001) covered the research objects of kindergarten pupils, higher graders of elementary schools, and high school students as well as the research fields of foreign language vocabulary memory, motivation to learn, mathematical problem solving, and reading comprehension of English and mathematics. Such research findings showed that multiple intelligences applied teaching activities could significantly enhance students' learning achievement, promote the motivation to learn, enhance reading the comprehension, and even enhance the ability of cooperative learning with peers. Broadly speaking, the framework of multiple intelligences cannot be promoted simply in kindergartens and elementary schools, but are suitable for high schools, universities, and even graduate schools or in-service training. A lot of international MBA courses are added creative thinking to strengthen the development of adaptability and creativity in the new era. For this reason, teaching with multiple intelligences to corporate employees' learning achievement and learning motivation is discussed in this study, expecting to help high-tech industry effectively well-utilize people's gifted uniqueness in the challenge of developing human resource potential.

## Literature Review

Simoncini et al. (2018) stated that teaching with multiple intelligences stressed on the provision of democratic, respectful, and multiple learning environment for each student being able to present the ability, self-affirm personal performance, and further induce strong learning interests to surpass the originally dominant intelligence field in learning outcome. Inan and Erkus (2017) indicated that using multiple intelligences for curriculum design could provide various intellectual learning activities and create the environment with which students were comfortable. Learning was the preparation for challenge; learners would develop by accepting challenges exceeding the current abilities. Encouraging students deeply and meaningfully to engage in the learned topics was the solid and durable learning basis for learning new affairs. The application of multiple intelligences and the creation of diverse classrooms to develop students' specialty allowed students maintaining learning motivation with active participation, building self-confidence, and developing self-motivation. Minnier et al. (2019) mentioned that the application of multiple intelligences to teaching was different from traditional teaching; teaching with multiple intelligences adopted multiple instruction strategies and activities. Many studies indicated that the application of multiple intelligences to teaching enhanced students' learning motivation and interests. The following hypothesis is therefore proposed in this study.

**H1**: Teaching with multiple intelligences would affect learning motivation.

Moncada and Mire (2017) indicated that teachers had to know each student's strengths and traits and appreciate individual advantages to give guidance and inspiration in order to strengthen the learning confidence. Multiple intelligences reminded teachers to comprehend and apply diverse teaching methods, transform existing curricula, or units into multiple learning opportunities, as well as carefully consider the taught concepts and confirm the most appropriate intelligence for communicating the content before planning curricula in order to ensure the achievement of proper teaching goals and promote students' learning achievement. Awang et al. (2017) proposed that teaching with multiple intelligences could positively enhance students' academic performance to make progress on English listening, speaking, reading, and writing. After applying multiple intelligences to English teaching, students enhanced learning achievement, learning interests, and learning motivation. Several researchers proposed that students appeared positive change on the learning achievement. Khong et al. (2017) indicated in the research results that higher-grader students in elementary schools being taught science based on multiple intelligences outperformed those receiving traditional teaching. According, the following hypothesis is proposed in this study.

**H2**: Teaching with multiple intelligences would affect learning achievement.

Russell et al. (2017) considered that the achievement of meaningful and effective learning to skillfully grasp the concept relied on students' intrinsic motivation, when students expected to acquire certain knowledge with e-learning. Khow and Visvanathan (2017) considered the value of e-learning that students could enhance learning achievement by acquiring good performance and presenting intrinsic motivation to contact broad professional knowledge/competence. Hunter and Hunter (2018) stated that students with high learning motivation presented more definite goals and strong desire to well-learn the learning content and showed higher expectation and better self-efficacy. It was also discovered that students with high learning motivation appear better performance, and students with intrinsic motivation outperformed those with extrinsic motivation. Consequently, the following hypothesis is proposed in this study.

**H3**: Learning motivation presents significantly positive effects on learning achievement.

## Methodology

### Measurement of Research Variable

#### (1) Teaching With Multiple Intelligences

Referring to Minnier et al. (2019), the following dimensions for the curriculum design of teaching with multiple intelligences, according to student needs, are proposed in this study.

1. Intrapersonal intelligence: Intrapersonal intelligence is defined as the intrapersonal ability according to individual self-knowing ability and self-perception to keenly and precisely perceive personal inner emotion, motivation, ability, intention, and desire.
2. Interpersonal intelligence: Intrapersonal intelligence is defined as being able to effectively perceive and discriminate others' emotion, affection, intention, feeling, motivation, and expectation as well as make proper responses to interpersonal relationship to further get along with people harmoniously.
3. Content-based curriculum: Content-based curriculum integrates knowledge and life, provides students with opportunities to apply knowledge, well-utilize community resources, and integrate community professional manpower for students learning with multiple intelligences and increasing learning channels.
4. Situated learning: Learning situations are co-constructed and maintained by teachers and students, are free, open, and cooperative, pay attention to overall conceptual knowledge orientation, and match students' sensory learning with teaching resources for learning in the real-life situation and respecting the difference in learners' learning outcome.

#### (2) Learning Motivation

According to the research of Cheng et al. (2018), students' learning motivation is divided into intrinsic learning motivation orientation and extrinsic learning motivation orientation in this study, as below.

1. Intrinsic orientation: containing favor of challenging courses, regarding learning as interest and hobby, considering that learning could expand vision, being able to actively learn new courses, learning for developing self-potential and realizing ideas.
2. Extrinsic orientation: covering learning for receiving others' affirmation, acquiring better performance, passing examinations or evaluation, showing off to others, competing with classmates, obtaining appreciation and attention from elders or the opposite sex, preventing from punishment and scold, avoiding the shame of failure, and entering ideal schools in the future.

#### (3) Learning Achievement

Referring to Zebari et al. (2018), the following dimensions for learning achievement are proposed in this study.

1. Learning effect-including test performance, time for completing schedule, and term performance.
2. Learning gain-containing learning satisfaction, achievement, and preference.

### Method and Model

Structural equation model is used as the research method in this study and Amos is utilized as the statistical tool. Structural equation model (SEM), also named covariance structure analysis, is used for analyzing causality model and precedes path analysis (PA), factor analysis, regression analysis, and analysis of variance. Structural equation model consists of two parts. The first part, measurement model, aims to construct the latent variable model with observed variables to understand the relationship between observed variables and latent variables; the constructed mathematical model is Confirmatory Factor Analysis (CFA). The second part, Structure Model, mainly discusses the causality among latent variables with path analysis, where observed variables are used; latent variables are used for Structure Model.

### Research Subject and Sampling Data

Aiming at employees in Southern Taiwan Science Park as the research objects, total 314 employees in high-tech industry are preceded the 30-week (2 h per week for total 30 h) experimental research. The questionnaire survey is preceded after the end of the 30-week course, and statistical methods are applied to test various hypotheses. Among the distributed 314 copies of questionnaire, 297 copies are valid, with the valid retrieval rate 95%.

### Reliability and Validity Test

Reliability and validity are important measurement standards. Merely the data results acquired from the questionnaire design with reliability and validity present the research value. AMOS is used for Confirmatory Factor Analysis (CFA) in this study, and SPSS 21 is applied to calculate the reliability and validity to test the questionnaire scale achieving the reliability and validity standard.

## Empirical Result

### Factor Analysis and Validity Analysis

Based on factor loadings, all items in this study are preceded confirmatory analysis. The factor loadings should be higher than 0.7; if not, the item does not show the representativeness and is removed. The Confirmatory Factor Analysis results show that all factor loadings of teaching with multiple intelligences, learning motivation, and learning achievement conform to the standard (>0.7), revealing high validity of the questionnaire scale.

Cronbach's α is used in this study for evaluating reliability; Cronbach's α higher than 0.7 achieves the reliability standard, and the ideal value should be higher than 0.9. Cronbach's α of teaching with multiple intelligences, learning motivation, and learning achievement in this study is higher than the suggested threshold and with the lowest value up to 0.8, revealing high reliability of the questionnaire scale.

### Test of Model Fit

“Maximum Likelihood” (ML) is utilized in this study for the estimation; the obtained Amos analysis results achieve convergence. The indicators standing for the external quality of model show (1) χ^2^ ratio = χ^2^ = 1.627, smaller than 3, (2) goodness-of-fit index GFI = 0.97, higher than 0.9 and adjusted goodness-of-fit index AGFI = 0.82, higher than 0.8, (3) root mean square residual RMR = 0.029, smaller than 0.05, and (4) incremental fit index 0.94, higher than 0.9. Overall speaking, the actual number of 297 samples is higher than the requirement for the basic number of samples, and the overall model fit indicators pass the test, fully reflecting good internal quality of the structural equation model.

Regarding the test of internal quality of structure, the squared multiple correlation (SMC) of manifest variables is higher than 0.5, revealing good measurement indicators of latent variables. Furthermore, latent variables of teaching with multiple intelligences, learning motivation, and learning achievement show the component reliability higher than 0.6 and the average variance extracted of dimensions is higher than 0.5, apparently meeting the requirement for the internal quality of model.

### Test of Path Relationship

**Figure 1:**
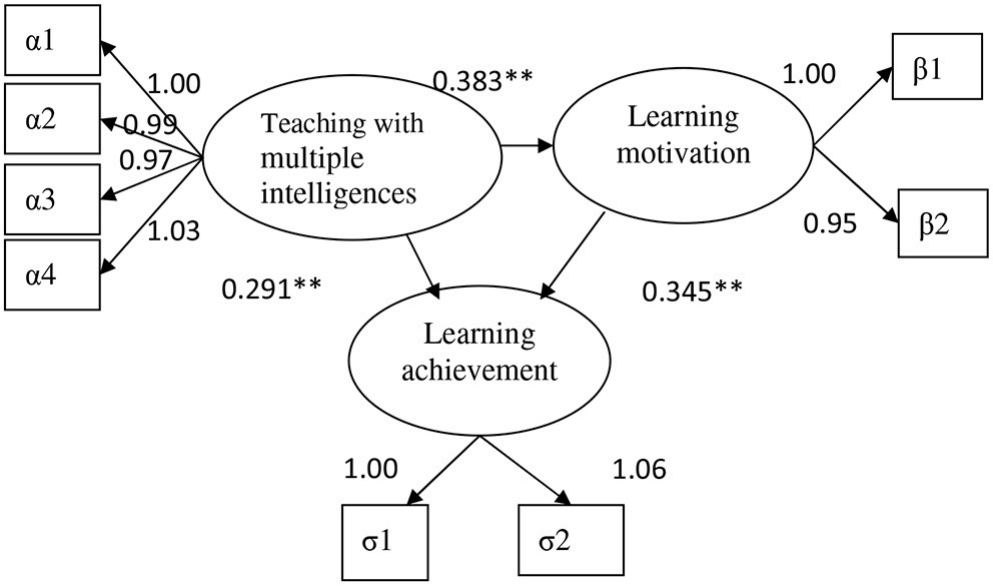
Path relationship.

Latent variables of intrapersonal intelligence, intrinsic orientation, and learning effect are regarded as the reference indicators with fixed 1. From the causality path in Table 1 and Figure 1, the estimates between other dimensions and variables appear significance. Interpersonal intelligence = 0.99 shows less explanatory power than intrapersonal intelligence, and learning gain = 1.06 presents better explanatory power than learning effect.

**Table 1:** Overall linear structure model analysis result.

**Factor dimension/evaluation standard**		**Estimate**
Teaching with multiple intelligences	Intrapersonal intelligence (α1)	1.00
	Interpersonal intelligence (α2)	0.99
	Content-based curriculum (α3)	0.97
	Situated learning (α4)	1.03
Learning motivation	Intrinsic orientation (β1)	1.00
	Extrinsic orientation (β2)	0.95
Learning achievement	Learning effect (σ1)	1.00
	Learning gain (σ2)	1.06
Teaching with multiple intelligences → Learning motivation		0.383^**^
Learning motivation → Learning achievement		0.345^**^
Teaching with multiple intelligences → Learning achievement		0.291^**^

** *p < 0.01*.

## Discussion

Teaching with multiple intelligences could effectively enhance the learning motivation of employees in high-tech industry to promote and continue the learning achievement. The research results are consistent with most of past research results (Ikiz and Cakar, 2010; Mahasneh, 2013). As Akkuzu and Akçay (2011) revealed, teaching with multiple intelligences was more effective than traditional teaching styles and such activities were interesting to facilitate students' interests in participation in course activities. In this case, the application of teaching with multiple intelligences allows employees in high-tech industry preceding learning activity with the advantageous intelligence to be more confident of learning challenges, rather than being inoculated to result in getting half the results with double efforts for learning with weaker intelligence, and further help promote the performance of organizational learning. The use of computers is inevitable for modern people; the use of ppt, films, or mv could properly attract the attention of employees in high-tech industry. Well-begun is half done; besides, computer-assisted teaching could largely assist employees in more difficult intelligence activity design, such as space, natural observer, and music intelligence. Teachers therefore should flexibly apply such resources. Moreover, teachers should take a long-term view, rather than focusing on immediate results. The cultivation of employees' active learning and high learning motivation would multiply and endure the learning validity.

## Conclusion

The research results reveal that Consistent with most past research results, it reveals that teaching with multiple intelligences indeed could effectively promote learning achievement and motivation to learn (Gardner and Hatch, 1989; Barrington, 2004; Akkuzu and Akçay, 2011). Employees in high-tech industry remarkably enhance learning achievement and learning motivation after the teaching with multiple intelligences. In this case, relevant academic competition could be held in organizations with proper rewards to effectively apply the employees' learning effectiveness and increase the learning motivation. Different from traditional teaching, teaching with multiple intelligences, with more personal practice and participation, allows employees in high-tech industry grasping the learning, rather than simply accepting knowledge. As a result, employees would enhance self-efficacy. For instance, employees in high-tech industry, under group learning, observation, and brainstorming, would make progress on reports, and learning comprehension as well as deepen and broaden learning motivation. Teachers, during the instruction, should praise and encourage for the progress of employees, create low-pressure, relaxing, and comfortable learning environment, and give more learning confidence to strength the learning motivation of employees in high-tech industry.

## Data Availability Statement

The original contributions presented in the study are included in the article/supplementary materials, further inquiries can be directed to the corresponding authors.

## Ethics Statement

The present study was conducted in accordance with the recommendations of the Ethics Committee of the Fuzhou Institute of Technology, with written informed consent being obtained from all the participants. All the participants were asked to read and approve the ethical consent form before participating in the present study. The participants were also asked to follow the guidelines in the form in the research. The research protocol was approved by the Ethical Committee of the Fuzhou Institute of Technology.

## Author Contributions

D-YL performed the initial analyses and wrote the manuscript. J-HC, C-MC, K-PH, and CJ assisted in the data collection and data analysis. All authors revised and approved the submitted version of the manuscript.

## Conflict of Interest

The authors declare that the research was conducted in the absence of any commercial or financial relationships that could be construed as a potential conflict of interest.

## Publisher's Note

All claims expressed in this article are solely those of the authors and do not necessarily represent those of their affiliated organizations, or those of the publisher, the editors and the reviewers. Any product that may be evaluated in this article, or claim that may be made by its manufacturer, is not guaranteed or endorsed by the publisher.
